# Efficacy of a chitosan tampon in the loop electrosurgical excision procedure: A prospective randomized controlled study

**DOI:** 10.1038/s41598-020-62965-1

**Published:** 2020-04-07

**Authors:** Gun Oh Chong, Yoon Hee Lee, Se Young Jeon, Hee-Young Yang, Sang-Hyun An

**Affiliations:** 1 Department of Obstetrics and Gynecology, School of Medicine, Kyungpook National University, Daegu, 41944 Republic of Korea; 2 Department of Obstetrics and Gynecology, Kyungpook National University Chilgok Hospital, Daegu, 41404 Republic of Korea; 3 Laboratory Animal Center, Daegu-Gyeongbuk Medical Innovation Foundation, Daegu, 41061 Republic of Korea

**Keywords:** Quality of life, Randomized controlled trials

## Abstract

It has been reported that chitosan has a hemostatic effect and an antibiotic activity. This study aimed to evaluate the efficacy and feasibility of using a chitosan tampon (Hemoblock-Tampon) in preventing hemorrhage and enhancing wound healing after the loop electrosurgical excision procedure (LEEP).This single-blind, prospective, randomized study included 62 consecutive patients who underwent LEEP for cervical intraepithelial neoplasia. A chitosan tampon (31 patients; treatment group), or a general tampon (31 patients; control group) was applied to the uterine cervix immediately after LEEP. One patient in the treatment group declined to participate in this study. Thus, 30 patients in the treatment group and 31 patients in the control group completed this study. For objective analysis of hemorrhage in the postoperative 2 weeks, the amounts of bleeding were checked daily with a pictorial blood assessment chart. We evaluated vaginal discharge, abdominal pain, and impairment in daily living during the postoperative 2 weeks using 5 visual analogue scale questionnaires.The bleeding count was significantly lower in the treatment group than in the control group (21.37 ± 16.86 vs. 40.52 ± 16.55, *p* = 0.0014). The sum of the scores of the 5 questionnaires was significantly lower in the treatment group than in the control group (6.53 ± 2.84 vs. 8.59 ± 2.88, *p* = 0.0079). The incidence of vaginal discharge was significantly lower in the treatment group than in the control group (20.0% vs. 48.4%, *p* = 0.0207). According to logistic regression, only the use of chitosan tampon reduced the risk of moderate to severe vaginal bleeding 2 weeks after surgery (Odd ratio, 0.213; 95% confidence interval, 0.06–0.76; *p* = 0.0172). Complete healing of the uterine cervix occurred in 86.7% of patients in the treatment group and in 61.3% of patients in the control group at 4 weeks after surgery (*p* = 0.0255).The use of chitosan tampons can reduce hemorrhage, vaginal discharge, abdominal pain, and impairment of daily living after LEEP. Moreover, chitosan tampon may help enhance wound healing.

## Introduction

The loop electrosurgical excision procedure (LEEP) is a method used for both the diagnosis and treatment of cervical intraepithelial neoplasia (CIN)^1^. This procedure has been widely used because it is an inexpensive, technically easy-to-perform procedure that requires only local anesthesia and has a low complication rate as well as good specimen quality^2,3^.

Despite these advantages, complications such as postoperative bleeding, abnormal vaginal discharge, abdominal pain, and infection have been reported^2–5^. These complications make patients anxious and cause interference in activities of daily living. Moreover, severe hemorrhage requires additional procedures and medical costs. The prevalence rate of LEEP complications was reported to range from 0.8% to 52%^5,6^.

Chitosan is a promising hemostatic agent because it can adhere to red blood cells and induce platelets to adhere, activate, and aggregate at the site of bleeding^7^. Furthermore, chitosan has biological properties such as hemostatic activity, antibacterial activity, and ability to accelerate wound healing^8^. Thereby, it is being used in many medical devices and health-care products^9^. Recently, several reports have shown the usefulness and effectiveness of chitosan for postpartum hemorrhage^10,11^. However, chitosan has not been used in the field of gynecologic surgery, especially after LEEP.

The objectives of this pilot study were as follows: (1) to examine the hemostatic efficiency and the reduction of blood clotting time by using an *in vitro* blood compatibility test; (2) to evaluate whether chitosan tampon could decrease postoperative bleeding, vaginal discharge, pain, and impairment of daily activities; and (3) to assess potential improvement in wound healing and treatment-associated complications such as hypersensitivity after LEEP.

## Results

### Comparison of blood clotting index (BCI) between chitosan gauze and general gauze

**Figure 1:**
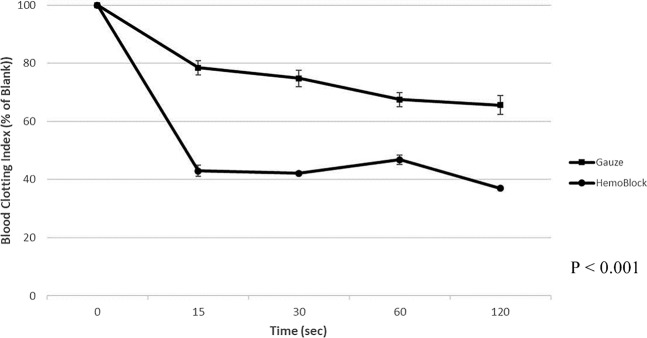
Measurement of *in vitro* blood clotting index.

The BCI was measured with reference to the anticoagulant citrate dextrose (ACD)-treated whole blood by using spectrometry. The mean BCIs were significantly lower for chitosan gauze than for general gauze (43.00 ± 1.94 vs. 78.45 ± 2.38, 15 s, *p* = 0.0026; 42.14 ± 0.55 vs. 74.80 ± 2.88, 30 s, *p* = 0.0024; 46.85 ± 1.64 vs. 67.52 ± 2.43, 60 s, *p* = 0.0009; and 36.92 ± 0.61 vs. 65.58 ± 3.22, 120 s, *p* = 0.0039). On the basis of the BCI results, blood coagulation was more effective with chitosan gauze than with general gauze (Fig. 1).

### Patients’ characteristics

The mean age was 43.40 ± 10.27 years in the treatment group and 46.06 ± 7.81 in the control group (*p* = 0.2576). There was no significant difference in terms of the proportion of menopausal women (23.3% vs. 32.3%, *p* = 0.4408). The final cervical pathology, and the size and depth of the surgical specimen were similar between the 2 groups (Table 1).

**Table 1:** Patients and pathologic characteristics.

Variables	Treatment group (n = 30)	Control group (n = 31)	*p*-Value
Age (years)	43.40 ± 10.27	46.06 ± 7.81	0.2576
Menopause status, n (%)	7 (23.3)	10 (32.3)	0.4408
LEEP histopathology			
≤LSIL	4 (13.3)	5 (16.1)	0.7602
≥HSIL	226 (86.7)	22 (83.9)	
Surgical specimen			
Size (cm^2^)	4.10 ± 1.36	4.63 ± 1.77	0.1940
Depth (cm)	1.55 ± 1.17	1.76 ± 1.10	0.4707

LEEP, loop electrosurgical excision procedure; LSIL, low-grade squamous intraepithelial neoplasia; HSIL, high-grade squamous intraepithelial neoplasia.

### Complications at 2 and 4 weeks after surgery

At the 2 weeks follow-up visit at the hospital, the sum of PBAC and patient questionnaires, degree of vaginal bleeding, and vaginal discharge were evaluated. The sum of PBAC and questionnaire scores during postoperative 2 weeks were significantly lower in the treatment group than in the control group (21.37 ± 16.86 vs. 40.52 ± 16.55, *p* = 0.0014; 6.53 ± 2.84 vs. 8.55 ± 2.88, *p* = 0.0079). The incidence of mild vaginal bleeding was similar between the 2 groups (16.7% vs. 19.4%, *p* = 0.7866); however, the incidence of moderate to severe vaginal bleeding was significantly lower in the treatment group than in the control group (13.3% vs. 41.9%, *p* = 0.0135). Moreover, the incidence of vaginal discharge was significantly lower in the treatment group than in the control group (20.0% vs. 48.4%, *p* = 0.0207).

At the follow-up visit at 4 weeks, vaginal bleeding, vaginal discharge, and healing status of the uterine cervix were evaluated. No significant differences were observed in vaginal bleeding and vaginal discharge between the 2 groups. However, the incidence of complete healing of the uterine cervix was significantly higher in the treatment group than in the control group (86.7% vs. 61.3%, *p* = 0.0255) (Table 2). No case of hypersensitive reaction occurred in both groups.

**Table 2:** Complications at 2 and 4 weeks after surgery.

Variables	Treatment group (n = 30)	Control group(n = 31)	*p*-Value
At 2 weeks			
Pictorial blood assessment chart	21.37 ± 16.86	40.52 ± 16.55	0.0014
Visual analogue scale score of 5 questionnaires	6.53 ± 2.84	8.55 ± 2.88	0.0079
Mild vaginal bleeding, n (%)	5 (16.7)	6 (19.4)	0.7866
Moderate or severe vaginal bleeding, n (%)	4 (13.3)	12 (41.9)	0.0135
Vaginal discharge, n (%)	6 (20.0)	15 (48.4)	0.0207
At 4 weeks			
Mild vaginal bleeding, n (%)	2 (6.7)	5 (16.1)	0.2503
Moderate or severe vaginal bleeding, n (%)	0 (0)	3 (9.7)	0.0831
Vaginal discharge, n (%)	0 (0)	1 (3.2)	0.3252
Complete healing of the cervix, n (%)	26 (86.7)	19 (61.3)	0.0255

### Logistic regression analysis for moderate to severe vaginal bleeding at 2 weeks after surgery

According to logistic regression, only the use of chitosan tampon reduced the risk of moderate to severe vaginal bleeding 2 weeks after surgery (OR, 0.213; 95% CI, 0.06–0.76; *p* = 0.0172). However, patient age, size and depth of the specimen, and final pathology were not associated with moderate to severe vaginal bleeding (Table 3).

**Table 3:** Logistic regression analysis for moderate to severe vaginal bleeding at 2 weeks after surgery.

	OR	95% CI	*p*-Value
Use of chitosan tampon (yes vs. no)	0.213	0.06–0.76	0.0172
Age (>45 years vs. ≤45 years)	2.064	0.66–6.44	0.2120
Size (>4 cm^2^ vs. ≤4 cm^2^)	2.400	0.72–7.96	0.1524
Depth (>1 cm vs. ≤1 cm)	1.661	0.50–5.54	0.4085
Pathology (≥HSIL vs. LSIL)	3.556	0.41–30.85	0.2499

OR, odds ratio; 95% CI, 95% confidence interval; HSIL, high-grade squamous intraepithelial neoplasia; LSIL, low-grade squamous intraepithelial neoplasia.

## Discussion

This study investigated the efficacy of chitosan tampon application after LEEP. Chitosan showed effective blood coagulation in the *in vitro* blood compatibility test. Furthermore, chitosan tampon was effective in reducing postoperative bleeding, vaginal discharge, and impairment of daily activities. Moreover, chitosan tampon may enhance the healing of the uterine cervix.

Chitosan has a positively charged surface and plays a role in attracting the negatively charged red blood cell membranes, resulting in hemagglutination^12^. Chitosan also induces platelet adhesion and activation and enhances platelet aggregation by absorbing plasma proteins and fibrinogen^13,14^. Another important property of chitosan that makes it a suitable material for preparation of wound dressing is its inherent antimicrobial activity. Free amine groups present in chitosan provide antimicrobial activity because they bind to the bacterial cell wall, thus causing bacterial cell lysis^15^. There are many commercially available chitosan-based composites for use in hemostasis and wound healing, including Chitogauze, Celox Gauze, Mini-sponge dressing, Hemcon, TraumaGauze, and ChitoFlex^16,17^. However, there are no commercially available products for LEEP.

Several methods for preventing hemorrhage after LEEP are described in the literature. Some studies have shown that routine prophylactic application of Mosel’s solution and local anesthesia with epinephrine could reduce postoperative vaginal bleeding^18,19^. Moreover, several hemostatic agents have been applied for reducing hemorrhage after LEEP; however, their effect on reducing hemorrhage remains controversial. Some studies demonstrated that Tissel (Baxter, Westlake Village, CA, USA) could reduce postoperative vaginal bleeding^20,21^. However, other reports showed that using hemostats such as Tachosil (Nycomen, Zurich, Switzerland) could not reduce postoperative vaginal bleeding^19,22^. Because most previous studies evaluated postoperative hemorrhage based on the subjective assessment of symptoms by patients and clinicians, exact evaluation of postoperative hemorrhage was difficult. Therefore, we used the PBAC to objectively measure hemorrhage during the postoperative 2 weeks. In this study, chitosan tampon significantly reduced postoperative vaginal bleeding (21.37 ± 16.86 vs. 40.52 ± 16.55, *p* = 0.0014).

Because LEEP is a relatively safe procedure, we also focused on self-estimated symptoms that can lead to disability in daily life, by using 5 visual analogue scale questionnaires. In this study, the sum of the scores of the 5 visual analogue scale questionnaires showed that vaginal discharge, abdominal pain, and impairment of daily activities could be reduced by chitosan tampon. Vaginal discharge and wound healing were also improved by chitosan tampon. The antimicrobial activity of chitosan may have influenced these results.

Chitosan has antiviral activity^23^. Moreover, a recent study reported that sulfated chitosan possesses broad anti-human papilloma virus (HPV) activities *in vitro* and may possibly inhibit HPV infection by targeting viral capsid protein and host phosphoinositide 2-kinase/Akt/mammalian target of rapamycin pathway^24^. Therefore, chitosan tampon may have potential as a novel anti-HPV agent. More comprehensive molecular studies should be performed to verify these findings. Furthermore, we plan to evaluate in the future whether the application of chitosan tampon affects disease recurrence and persistence of HPV infection after LEEP.

Our study has some limitations. First, it is a pilot study with a limited number of patients. Second, this study was conducted in a single-blind setting because we could not produce an adequate placebo control.

Despite these limitations, our study offers some unique and significant findings. This is the first study to evaluate the usefulness of chitosan application after LEEP. Moreover, we evaluated postoperative hemorrhage, vaginal discharge, abdominal pain, and impairment of daily activities by using the patients’ self-estimated visual score to reduce bias.

In conclusion, chitosan tampon is effective in reducing vaginal bleeding, vaginal discharge, abdominal pain, and impairment of daily living after LEEP. Moreover, chitosan tampon may help enhance wound healing. However, further studies with a large number of patients should be performed to confirm our findings.

## Materials and Methods

### Measurement of BCI *in vitro*

**Figure 2:**
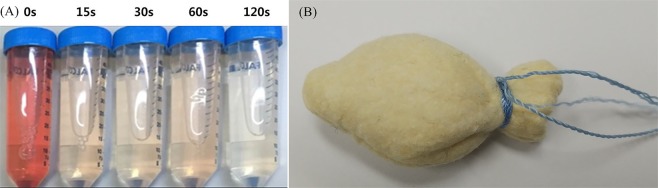
*In vitro* blood compatibility test. (**A**) Chitosan tampon (Hemoblock-Tampon).

A chitosan gauze and a general gauze of 2 × 2 cm size and 2 mm thickness were placed in a test tube with a flat base. The test tube was boiled in a water bath with the automatic temperature controller set at 37 °C for 5 min. Then, dripping of blood was done carefully to ensure that the surface would be completely covered with 0.27 mL of human blood (whole blood treated with 0.3 mL ACD and added with 0.024 mL calcium chloride). The test tube containing blood was incubated in an incubator with the automatic temperature controller set at 37 °C for 10 min. A 10 mL volume of deionized distilled water was carefully dripped to dissolve the coagulated blood components (Fig. 2A). Subsequently, a 10 mL dissolver contained in a test tube was centrifuged at 100 g for 30 s. Following the centrifugation, the supernatant was placed in a glass tube containing 40 mL deionized distilled water and maintained at 37 °C for 60 min. A blood clotting test was performed according to the relative absorbance measured at a wavelength of 542 nm on a spectrometer. It was hypothesized that the reference absorbance value might be 100 at a wavelength of 542 nm for the solution mixture of 50 mL deionized distilled water and 0.25 mL ACD-treated whole blood. The BCI was measured following previously reported methods, and calculated by the following equation^25^.

$${\rm{BCI}}=100\times ({\rm{Absorbance}}\,{\rm{of}}\,{\rm{sample}})/({\rm{Absorbance}}\,{\rm{of}}\,{\rm{reference}})$$

### Study population

After obtaining approval from the institutional review board of Kyungpook National Univeristiy Chilgok hospital, we recruited the study population. The inclusion criteria were age 20–65 years, biopsy-confirmed CIN grade ≥2, not pregnant status, and provision of informed written consent for participation. Patients with known hypersensitivity to crustacean foods were excluded.

The sample size was estimated, assuming a hypothetical 30% higher overall complication rate in patients who underwent LEEP without the chitosan tampon (control group) compared with those in whom a chitosan tampon was applied (treatment group; 80% power; type I error probability, 0.05; drop rate, 10%). Theoretically, 31 patients were required for each group. Between December 2017 and September 2018, a total of 62 patients underwent LEEP with or without chitosan tampon application. Among these patients, 1 patient in the treatment group declined to participate in this study. Finally, 61 patients were recruited (30 in the treatment group and 31 in the control group) and assigned to LEEP with or without chitosan tampon application.

### Chitosan tampon

Chitosan tampon (Hemoblock-Tampon; Incore, Daegu, Korea) contains chitosan fiber, cotton, barium sulfate, and polyamide. The shape of the chitosan tampon was designed for fixing to the wound after LEEP (Fig. 2B).

### Study design

This was a single-blind, prospective, randomized study involving 62 consecutive patients undergoing LEEP for biopsy-confirmed CIN grade ≥2. Our committee approved the research and confirmed that all research was performed in accordance with relevant gauidelines and regulation. Informed consent was obtained from all participants. The enrolled patients were randomly allocated (1:1) to a treatment group or control group using a randomized table prepared using a random sequence generator (www.random.org). Among these patients, 61 (30 in the treatment group and 31 in the control group) completed follow-up. The present study was registered at Clinical Research Information Service (CRIS, https://cris.nih.go.kr, KCT0003696, resgisted on April 1, 2019). The study was informed by the CONSORT approach and the results were reported accordingly. The authors confirm that all ongoing and related trials for this intervention are resistered.

All procedures were performed using a right-angled loop carrying high-frequency current (High Frequency; Sometech, Seoul, Korea) with as single pass. We did not use any hemostatic agents during or after LEEP, such as epinephrine or Mosel solution in both groups. Before tampon application, hemostasis was done completely using an electrocautery device. Chitosan tampon was applied to the uterine cervix immediately after LEEP surgery in the treatment group, and general tampon made of cotton was applied to the uterine cervix in the control group for 12 hours. Pateints removed tompons by themselves at home.

**Figure 3:**
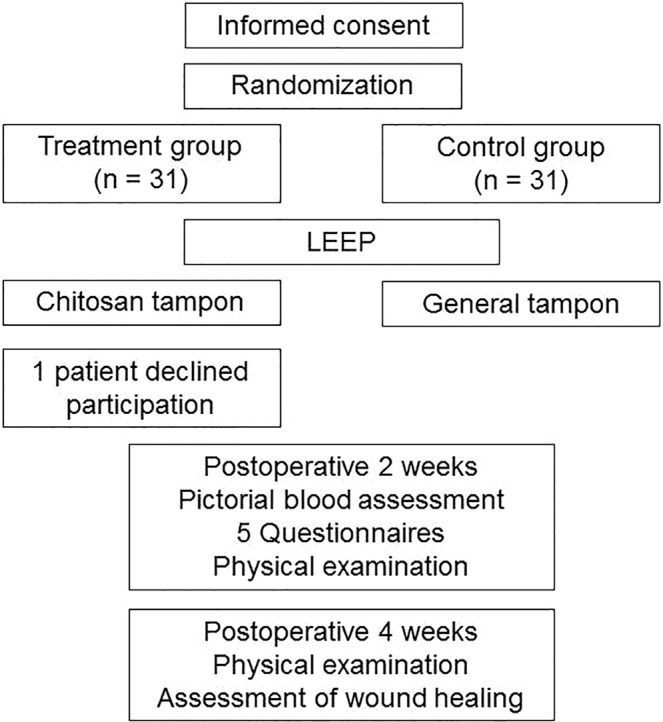
Study design.

Clinical follow-up was performed at 2 and 4 weeks after LEEP. For objective and exact measurement of postoperative bleeding, vaginal discharge, abdominal pain, and impairment of daily living, we used a pictorial blood loss assessment chart (PBAC) and 5 visual analogue scale questionnaires during the 2 weeks after surgery (Supplementary Table 1). When patients returned to the outpatient clinic 2 and 4 weeks after surgery, we evaluated the wound healing process and checked for vaginal bleeding (and determined whether it required intervention) and vaginal discharge. Mild vaginal bleeding was defined as no requirement for intervention, moderate vaginal bleeding as the requirement for intervention (chemical cautery and/or electrocautery) at the outpatient clinic, and severe vaginal bleeding as a visit to the emergency department. Complete wound healing was defined as the appearance of a normal cervix without discharge and bleeding. The details of the study design and protocol are summarized in Fig. 3.

### Statistical analysis

Continuous data were expressed as mean ± standard deviation and categorical data as frequency and percentage. Differences between subsets were evaluated with Student’s *t*-test, and differences between proportions were compared with the chi-square test or Fisher’s exact test. A logistic regression model was used to evaluate clinical variables for moderate and severe vaginal bleeding 2 weeks after surgery, and estimated odds ratios (ORs) with 95% confidence intervals (95% CIs) were presented.

All statistical tests were 2-sided, and a *p*-value of <0.05 was considered significant. Statistical analysis was performed using SPSS software version 22.0 (SPSS, Chicago, IL, USA) and Medcalc version 15.4 (Medcalc Software, Ostend, Belgium).

## Supplementary information

Supplementary table 1.

Supplementary Dataset 1.
