# Supplementary material for: Efficacy of a chitosan tampon in the loop electrosurgical excision procedure: A prospective randomized controlled study
Source: Sci Rep. 2020 Apr 7;10:6017. doi: 10.1038/s41598-020-62965-1 (PMC7138841; doi:10.1038/s41598-020-62965-1)
Supplement: Supplementary file 1 — Supplementary table 1. [file 41598_2020_62965_MOESM1_ESM.pdf]

Efficacy of a chitosan tampon in the loop electrosurgical excision procedure: A prospective randomized controlled study

Gun Oh Chong[1,2,*,+] , Yoon Hee Lee[1,2+] , Se Young Jeon[2] , Hee-Young Yang[3],

and Sang-Hyun An[3]

[1]Department of Obstetrics and Gynecology, School of Medicine, Kyungpook National University, Daegu, 41944, Republic of Korea

[2]Department of Obstetrics and Gynecology, Kyungpook National University Chilgok Hospital, Daegu, 41404, Republic of Korea

[3]Laboratory Animal Center, Daegu-Gyeongbuk Medical Innovation Foundation, Daegu, 41061, Republic of Korea

[*]corresponding author gochong@knu,ac,kr

[+]these authors contributed equally to this work as co-first authors

Supplementary table 1. Five questionnaires for evaluating vaginal discharge, impairment of daily living, and abdominal pain using the visual analogue scale.

Questionnaires	None of the time	A little of the time	Some of the time	Most of the time	All of the time
Interfered with your physical activity?	1	2	3	4	5
Made you concerned about soiling your underwear?	1	2	3	4	5
Made you feel that it was difficult to carry out your usual activities?	1	2	3	4	5
Interfered with your social activities?	1	2	3	4	5
How much bodily pain have you had?	1	2	3	4	5
